# Community-wide outbreaks of haemolytic uraemic syndrome associated with Shiga toxin-producing *Escherichia coli* O26 in Italy and Romania: a new challenge for the European Union

**DOI:** 10.2807/1560-7917.ES.2016.21.49.30420

**Published:** 2016-12-08

**Authors:** Ettore Severi, Flavie Vial, Emilie Peron, Otilia Mardh, Taina Niskanen, Johanna Takkinen

**Affiliations:** 1 European Centre for Disease Prevention and Control (ECDC), Stockholm, Sweden; 2 European Programme for Intervention Epidemiology Training (EPIET), European Centre for Disease Prevention and Control (ECDC), Stockholm, Sweden; 3 Gastrointestinal, zoonosis and tropical diseases unit, Department of infectious diseases epidemiology, Robert Koch Institute, Berlin, Germany

**Keywords:** haemolytic uraemic syndrome, Shiga Toxin-producing Escherichia coli, Verocytotoxin-producing Escherichia coli, STEC O26, VTEC O26, outbreak

**To the editor:** In their recent article in *Eurosurveillance*, Germinario et al. describe a community-wide outbreak of Shiga toxin 2-producing *Escherichia coli* (STEC) O26:H11 infections associated with haemolytic uraemic syndrome (HUS) and involving 20 children between 11 and 78 months of age in southern Italy during the summer 2013 [1]. The investigation identified an association between STEC infection and consumption of dairy products from two local milk-processing establishments. We underline striking similarities to a recent multi-country STEC O26 outbreak in Romania and Italy and discuss the challenges that STEC infections and their surveillance pose at the European level.

In March 2016, Peron et al. published, also in *Eurosurveillance*, early findings of the investigation of a community-wide STEC infection outbreak in southern Romania [2]. As at 29 February 2016, 15 HUS cases with onset of symptoms after 24 January 2016, all but one in children less than two years of age, had been identified, three of whom had died. Aetiological confirmation was retrospectively performed through serological diagnosis and six cases were confirmed with STEC O26 infection. Shortly after this publication, and following the identification of the first epidemiologically-linked case in central Italy, the European Centre for Disease Prevention and Control (ECDC) and the European Food Safety Authority (EFSA) published a joint Rapid Outbreak Assessment [3]. The Italian and Romanian epidemiological, microbiological and environmental investigations implicated products from a milk-processing establishment in southern Romania as a possible source of infection. The dairy plant exported milk products to at least four European Union (EU) countries. The plant was closed in March 2016 and the implicated food products recalled or withdrawn from the retail market.

Pulsed Field Gel Electrophoresis (PFGE) and whole genome sequencing (WGS) analyses did not establish a microbiological link between the Italian (2013) and the Romanian/Italian (2016) outbreaks (personal communication, Stefano Morabito, October 2016). However, the epidemiological similarities between the two community-wide outbreaks associated with HUS and STEC O26 infections, mostly affecting young children and implicating dairy products, are notable. While raw milk and unpasteurised dairy products are well known potential sources of STEC infection, milk products, as highlighted by Germinaro *et al*. [1], have been rarely implicated in community-wide STEC outbreaks in the past, emphasising an emerging risk of STEC O26 infection associated with milk products.

Reporting of STEC O26 infections has been steadily increasing in the EU since 2007, partly due to improved diagnostics of non-O157 sero-pathotypes [4]. The attention to non-O157 STEC sero-pathotypes rose considerably after the severe STEC O104 outbreak that took place in Germany and France in 2011 during which almost 4,000 cases and more than 50 deaths were reported [5]. In light of the recently published outbreaks related to dairy products and the simultaneous increased reporting of isolations of STEC O26 from milk and milk products in the EU/European Economic Area (EEA) [6], strengthening STEC surveillance in humans and food and enhancing HUS surveillance in children less than five years of age is warranted. Paediatric nephrologists should be sensitised to this effect and further joint studies between food and public health sectors be increased.
